# Can Bcl-XL expression predict the radio sensitivity of Bilharzial-related squamous bladder carcinoma? a prospective comparative study

**DOI:** 10.1186/1471-2407-11-16

**Published:** 2011-01-12

**Authors:** Ahmad M Abdel Raheem, Diaa A Hameed, Ehab O ElGanainy, Eman Mosad, Mostafa E Abdelwanis, Nermen A Kamel, Hisham M Hammouda, Mohammad A Abdelaziz, Khaled Hemeyda

**Affiliations:** 1 Urology Department, Assiut University Hospital, Assiut, Egypt; 2 Urology Department, Faculty of medicine, Assiut University, Assiut, Egypt; 3 Immunohistochemistry Laboratory, Clinical Pathology Department, South Egypt Cancer Institute, Assiut University, Assiut, Egypt; 4 Radiotherapy Department, South Egypt Cancer Institute, Assiut University, Assiut, Egypt; 5 Pathology Department, Faculty of Medicine, Assiut University, Assiut, Egypt; 6 Public Health Department, Faculty of Medicine, Assiut University, Assiut, Egypt

## Abstract

**Background:**

Local pelvic recurrence after radical cystectomy for muscle invasive bilharzial related squamous cell carcinoma accounts for 75% of treatment failures even in organ confined tumors. Despite the proven value of lymphadenectomy, up to 60% of patients undergoing cystectomy do not have it. These factors are in favor of adjuvant radiotherapy reevaluation. objectives: to evaluate the effect of adjuvant radiotherapy on disease free survival in muscle invasive bilharzial related squamous cell carcinoma of the urinary bladder and to test the predictability of radio-sensitivity using the anti apoptotic protein Bcl-XL.

**Methods:**

The study prospectively included 71 patients, (47 males, 24 females) with muscle invasive bilharzial related squamous cell carcinoma of the bladder (Stage pT2a-T3N0-N3M0) who underwent radical cystectomy in Assiut university hospitals between January 2005 and December 2006. Thirty eight patients received adjuvant radiotherapy to the pelvis in the dose of 50Gy/25 fractions/5 weeks (Group 1), while 33 patients did not receive adjuvant radiotherapy (group 2). Immunohistochemical characterization for bcl-xL expression was done. Follow up was done every 3 months for 12 to 36 months with a mean of 16 ± 10 months. All data were analyzed using SPSS version 16. Three years cumulative disease free survival was calculated and adjusted to Bcl-XL expression and side effects of the treatment were recorded.

**Results:**

The disease free cumulative survival was 48% for group 1 and 29% for group 2 (log rank p value 0.03). The multivariate predictors of tumor recurrence were the positive Bcl-XL expression (odd ratio 41.1, 95% CI 8.4 - 102.3, p < 0.0001) and radiotherapy (odd ratio 0.19, 95% CI 0.05 - 0.78, p < 0.02). With Cox regression, the only independent multivariate predictor of radio-sensitivity was the Bcl-XL expression with odd ratio 4.6 and a p value < 0.0001. All patients tolerated the treatment with no life threatening or late complications during the period of follow up.

**Conclusions:**

Adjuvant radiotherapy for muscle invasive bilharzial related squamous cell carcinoma of the urinary bladder has potential effectiveness and minor side effects. Moreover, Bcl-XL expression is a valuable tool for predicting those who might not respond to this adjuvant treatment.

## Background

Bilharzial related squamous cell carcinoma (SCC) occurs commonly in the Middle East, South-east Asia and South America where schistosomiasis is endemic [1]. In Egypt, SCC represents nearly 60 - 67.6% of all bladder cancers and it usually presents as a muscle invasive tumor [2,3]. Local pelvic recurrence after radical cystectomy accounts for about 75% of treatment failures even in those with organ confined tumors. This high incidence of local recurrence highlights the necessity of using adjuvant therapy (AR) to improve the final treatment outcome [4]. The use of AR after cystectomy has long lost favor within the oncology community including the radiation oncologists. Yet, many oncologists still believe in the idea of "sterilizing" the pelvis by AR [5]. The beneficial anti-cancer effects of radiotherapy are predominantly mediated through induction of apoptosis in tumor cells, de novo or as a result of induced damage of cellular metabolic processes or cell cycle control mechanisms. Accordingly, it is possible that tumors which exhibit apoptosis may be more sensitive to radiotherapy [6]. Bcl-2 family protein expression modulates response to radiotherapy where pro-apoptotic members enhance radio-sensitivity while anti-apoptotic members like Bcl-XL enhance radio-resistance [7]. Treatment-refractory cancers from a variety of sites display high expression of the pro-survival (anti-apoptotic) Bcl-XL factor [8] which suggests that Bcl-XL participates in multimodality resistance to radiation [9,10]. Bcl-XL is a negative prognostic factor in muscle invasive bilharzial related SCC (MIBR-SCC) of the bladder [11].

To our knowledge no data could be found in the available literature regarding the importance of Bcl-XL expression as a predictor of the response to AR among bilharzial or non- bilharzial bladder cancer patients. It would be advantageous to know forehand whether a particular patient will get any benefit in face of the potential harm due to radiotherapy.

The aim of this prospective study is to test the potential benefit of AR and the ability of Bcl-XL expression to predict the response to AR in patients with MIBR-SCC of the bladder.

## Methods

The current study included 71 patients, (47 males and 24 females) with histologically proven MIBR-SCC of the bladder (Stage pT2a-T3N0-N3M0) of different grades (grade 1 to 3).

All patients underwent radical cystectomy for male patients and anterior pelvic exenteration for female patients in the period from January 2005 to December 2006 in the South Egypt Cancer Institute and the department of urology, faculty of medicine, Assuit University. The presence of Schistosoma haematobium eggs in the cystectomy specimens was an inclusion criterion.

Every patient was offered the information about the effects, benefits and possible side effect of adjuvant radiotherapy. The patients were free to choose to be treated according to the international routine treatment of bladder SCC which is radical cystectomy alone, or to be subjected to the routine of our institute, which is radical cystectomy followed by radiotherapy.

Among those, 38 patients (group 1) agreed to receive AR to the pelvis 3 weeks after cystectomy. AR was given in the dose of 50Gy/25 fractions/5 weeks using linear accelerator (Siemens Mevatron) 6 - 15 MV Photons at 100 cm source axis distance. The upper border of the target was the junction of L5 - S1 vertebrae, the lower border was the lower margin of the obturator foramen. The lateral borders were 1.5 cm lateral to the pelvic brim, the anterior border was the anterior of the symphysis pubis and the posterior border included the anterior one third of the rectum. Three fields' technique (one anterior and 2 lateral wedged fields) was used. This arrangement is supposed to give a homogenous distribution to the target volume. The remaining 33 patients (group 2) refused AR based on different social and cultural backgrounds, such as how far they lived from the institute (sometimes more than 500 KM) and some prevalent exaggerated concerns about the side effects of radiotherapy, sometimes potentiated by personal previous experience of a relative of the patient.

Study scientific approval was obtained from the urology department scientific board and ethically approved by the ethics committee of the Faculty of Medicine, Assiut University (that conforms to the provisions of the Declaration of Helsinki). Informed written consents were obtained from all patients before enrollment in the study with guarantee of confidentiality.

Formalin fixed, paraffin embedded tissue from parts of the cystectomy specimens from all 71 patients were used for ordinary histopathological studies. Immunohistochemical characterization for Bcl-XL expression was done by the indirect immuno-peroxidase technique using the kit produced by LAB VISION Corporation. It is supplied as total IgG purified from rabbit anti-serum by protein A chromatography. It is prepared at 1 mg/ml in 10 mM PBS (Phosphate-buffered saline), ph 7.4.

### Staining Protocol

Tissue sections (4 μm thick) were cut immediately before staining and were heated to 56°C for 20 minutes. Protein blocking was accomplished through application of Ultra V block for 5 minutes and application of 5% normal Goat serum for 30 minutes. The primary antibody was applied in a concentration of 1:400 and was incubated in a moist humidity chamber overnight. Biotinylated Goat Anti-polyvalent was then applied and incubated for 10 minutes at room temperature. Streptavidin Peroxidase was then applied and incubated for 10 minutes at room temperature. One drop (40 μl) of Diaminobenzidine Chromogen was added to 1 ml of Diaminobenzidine Substrate (mixed by swirling) and the mixture was then applied to the tissue sections and incubated for 5 minutes. The sections were counterstained with Mayer's hematoxylin for few seconds.

Tissue samples with known expression for the marker (colon carcinoma stained with anti- Bcl-XL) served as positive control. Negative controls were sections treated as described above, but with the primary antibody replaced with pooled non-immune mouse IgG of the same concentration.

All sections were analyzed with a BX-40 bright field microscope under X10-20 objectives. When questions arouse concerning tissue morphology, H&E-stained sections were reviewed for confirmation. Staining was classified as negative when no or weak (less than 20%) expression of the anti-apoptotic marker Bcl-XL was observed within the cytoplasm of tumor cells. Staining was classified as positive when significant uniform cytoplasmic expression of the anti-apoptotic marker Bcl-XL (greater than 20%) was observed within the cytoplasm of tumor cells.

Follow up of all patients for 3 years after cystectomy or until recurrence had occurred was done every 3 months and included clinical examination, serum creatinine, alkaline phosphatase, abdominal ultrasonography and chest x-ray. Abdominal computerized tomography and bone scans were done when findings suggested disease progression, defined as any emerging local or distant tumor.

### Statistical analysis

All data were analyzed using SPSS (Statistical Program for Social Sciences version 16 for windows, 2001, SPSS Inc., Chicago, IL, USA). Univariate analysis was done using chi-square test with Fisher's exact correction and independent T test. Multivariate analysis was done using Logistic regression analysis to identify the most important risk factor for recurrence, the Kaplan Meier survival analysis with log rank p value was done to compare the effect of radiotherapy on recurrence. The effect of radiotherapy was adjusted to age group (older or younger than 50 years), sex, tumor size (≥ or <3 cm), stage (≥ or < stage III), grade, lymph node involvement and Bcl-XL expression as univariate predictors. Cox regression was done to test the significant factors as multivariate predictors of the efficacy of AR. *P *value < 0.05 was considered to be significant.

## Results

Table 1 shows the tumor characteristics in both treatment groups, there was no significant difference between both groups as regards clinico-pathological characteristics.

**Table 1:** Patients' clinicopathological data

		Total		AR		AR not done		
			
		N (71)	%	N (38)	%	N (33)	%	P -value
Age (Ys)	<50	37	52.1%	22	57.9%	15	45.5%	0.2
		
	≥50	34	47.9%	16	42.1%	18	54.5%	

Sex	Male	47	66.2%	24	63.2%	23	69.7%	0.4
		
	female	24	33.8%	14	36.8%	10	30.3%	

Tumor size	Small (<3 cm)	23	32.4%	9	23.7%	14	42.4%	0.07
		
	Large (≥3 cm)	48	67.6%	29	76.3%	19	57.6%	

Stage	T 2a	11	15.5%	7	18.4%	4	12.1%	0.5
		
	T 2b	50	70.4%	27	71.1%	23	69.7%	
		
	T 3	7	9.9%	2	5.3%	5	15.2%	
		
	T 4a	3	4.2%	2	5.3%	1	3.0%	

Grade	1	25	35.2%	16	42.1%	9	27.3%	0.09
		
	2	32	45.1%	18	47.4%	14	42.4%	
		
	3	14	19.7%	4	10.5%	10	30.3%	

LN	+ve	10	14.1%	3	7.9%	7	21.2%	0.3
		
	-ve	39	54.9%	23	60.5%	16	48.5%	
		
	Missing data	22	31.0%	12	31.6%	10	30.3%	

Bcl-XL	-ve	42	59.2%	23	60.5%	19	57.6%	0.5
		
	+ve	29	40.8%	15	39.5%	14	42.4%	

AR: Adjuvant radiotherapy; LN: Lymph nodes; +ve: positive; -ve: negative.

Using recurrence as a dependent factor, comparing the 2 groups (recurrence and no recurrence) concerning different predictors associated with recurrence, table 2 showed no statistical difference between the 2 groups except for Bcl-XL expression, tumor grade and AR. Recurrence occurred in 89.7% and 23.8% among patients with positive and negative Bcl-XL expression respectively (*p*-value < 0.0001). Recurrence was more associated with grade 2 and 3 tumors (59.4%, 78.6% respectively) with a p-value 0.002. Recurrence was noted in 39.5% of patients who were offered AR while in 63.6% of patients who were not offered AR (p-value 0.04).

**Table 2:** Univariate analysis for Factors associated with recurrence:

		Recurrence		
			
Variable		Yes (n = 36)	No (n = 35)	p-value
Age (mean ± SD)		48.8 ± 10.03	45.8 ± 9.22	NS**

Sex	Male	22(46.8%)	25 (53.2%)	NS*
		
	Female	14 (58.3%)	10 (41.7%)	

Bcl-XL expression	-ve	10 (23.8%)	32 (76.2%)	0.000*
		
	+ve	26 (89.7%)	3(10.3%)	

Histo-pathologic Grade	Grade 1	6 (24%)	19 (76%)	0.002*
		
	Grade 2	19(59.4%)	13(40.6%)	
		
	Grade 3	11(78.6%)	3(21.4%)	

Radiotherapy	Done	15 (39.5%)	23 (60.5%)	0.04*
		
	Not Done	21 (63.6%)	12 (36.4%)	

Stage	Stage 1-2	31(50.8%)	30(49.2%)	NS*
		
	Stage 3	5 (50%)	5(50%)	

Tumor Size	Small < 3 cm	9(39.1%)	14(60.9%)	NS*
		
	Large > 3 cm	27 (56.2%)	21(43.8%)	

** Independent T test, * Fisher Exact test.

Using a stepwise logistic regression to identify the most predicting factors associated with recurrence, positive Bcl-XL expression was the most significant factor (Odd's ratio 41.1, 95% confidence interval (CI): 8.4 - 102.3 and *p *- value 0.000) followed by AR which was a protective factor (Odd's ratio 0.19, 95% CI: 0.05 - 0.78 and *p *- value 0.02) (table 3)

**Table 3:** Logistic regression analysis for Factors predicting recurrence:

Variables	Wald	p-value	Odds Ratio	95% C.I	
				
				Lower	Upper
Radiotherapy Done	5.317	0.02	0.19	0.05	0.78

Positive Bcl-XL expression	20.907	0.000	41.1	8.4	102.3

R2	0.60				

With Cox regression analysis, the only independent multivariate predictor of radio-sensitivity was the Bcl-XL expression with odd ratio 4.6, 95% CI 2.2 - 9.6 and a p value < 0.0001.

**Figure 1:**
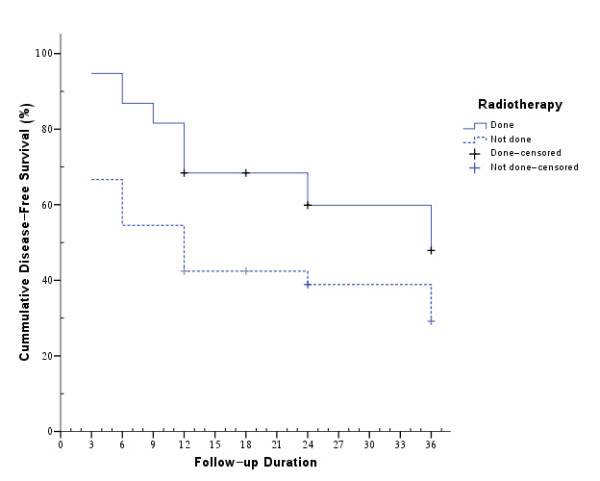
**Kaplan Meier Survival analysis for the effect of radiotherapy on cumulative disease free survival **.

After 3 years follow up the disease free cumulative survival was 48% for those who received AR (mean disease free survival of 26 ± 2 months; 95% CI: 22 - 31 months, median survival 18 months) while it was only 29% for those who did not (mean disease free survival of 18 ± 3 months; 95% CI: 13 - 23 months, median survival 12 months) (log rank p value 0.03) (Figure 1).

All the patients tolerated the treatment protocol with no life threatening toxicity or complications and in no patients the radiotherapy schedule had to be interrupted due to side effects. According to the national cancer institute classification of common toxicity [12], the side effects were in the form of diarrhea grade 1(increase of <4 stools/day) in 11(29%) and diarrhea grade 2 (increase of 4-6 stools/day or nocturnal stools) in 10 (26%) patients. All patients responded to simple anti-diarrheal treatment within one month. No late complications (including intestinal obstruction) were encountered in our patients in the period of follow up.

## Discussion

In this study 71 patients had radical cystectomy (or anterior pelvic exenteration) for MIBR-SCC of the bladder. Thirty eight patient received AR, while 33 refused it. A lot of cultural, and socioeconomic factors affected the patients' choice, and although we couldn't just allocate the patient into treatment groups because we could neither deny the patient a chance of a possible beneficial treatment (based on our experience), nor we could force the patient to comply with our routine which is still controversial. Yet, this still appears as a limitation to the study as it is not a formal way to randomize patients between treatment groups.

The 3 years mean disease free survival was 26 months for patients who received AR and 18 months for those who did not. Fifty percent of the relapses in the "no radiotherapy" group occurred within 3 months after the operation, in our opinion this supports the concept of gaining more local control via AR. The early recurrence that would occur within 3 months of the operation seems to be prevented by AR. Afterwards, the rates are not very different from the AR group, however, the recurrence rate is much less in the AR group because there is much less recurrences within the first 3 months.

These results are further support to those obtained by other investigators who valued the use of AR for MIBR-SCC of the bladder.

In a controlled randomized study, Zaghloul et al. compared cystectomy alone with cystectomy plus AR, using two different fractionation schedules. The 5-year disease free survivals were 49% and 44% in the two AR groups respectively, compared to 25% in the radical cystectomy-alone group [13].

In spite of the small sample size, the results of our study support the case for AR in MIBR-SCC of the bladder. The rationale behind this practice is multifactorial. Looking at the patterns of recurrence after cystectomy, large surgical series suggest that local failures with or without distant metastasis occur in about 13% to 30% of all patients [14]. This percentage reaches 75% in case of MIBR-SCC [3]. Another factor is the wide variation of the doctors' practice towards lymph node dissection during cystectomy. Although lymphadenectomy has a proven value in decreasing recurrence after cystectomy, it seems that up to 60% of patients going through cystectomy do not have lymphadenectomy [15]. Moreover, the effect of radiation on muscle invasive bladder cancer in many bladder preservation protocols, which can be equally curative to cystectomy, is well appreciated [16].

These factors are in favor - in our opinion - of the use of AR. Yet, this adjuvant therapy shouldn't be promoted as a replacement of lymphadenectomy.

The argument against AR is based on studies like Reisinger et al. who reported that bowel obstruction occurred in 37% of patients who received AR compared with 8% in those who did not [17].

The use of fractionated dose techniques and new technologies like the high-energy linear accelerator and computed tomography planning that deliver the maximum dose to the area of interest with minimum radiation hazard to the bowel decreases the rate of chronic bowel complications to 12% [18].

In the current series acute bowel complications after AR occurred in 21(55%) patients in the form of diarrhea grade 1:2 according to the National Cancer Institute classification [12]. No patient had intestinal obstruction as a chronic complication of the treatment. The overall advantage of AR as regards disease free survival in this study was obvious.

Bcl-XL expression was the only significant predictor of radio resistance. An explanation of this relationship between Bcl-XL over-expression and radio-resistance is that Bcl-XL resides in the outer mitochondrial membrane inhibiting the apoptotic process through which radiotherapy affects tumor cells [19]. Also, it was presumed that Bcl-XL confers radio-resistance by abrogating mitochondrial cytochrome-C release and activation of caspase-3 [20]. This relation between Bcl-XL over-expression and radio-resistance opens the way towards better selection of the cases, hence getting the best results as regards cancer control while avoiding the possible side effects of radiotherapy.

To our knowledge no comparable data could be found in the available literature regarding the importance of Bcl-XL expression as a predictor of the response to AR among bilharzial or non- bilharzial bladder cancer patients. However, the value of other anti apoptotic markers' expression (e.g. Bcl-2 and p53) in determining radio-sensitivity of bladder cancer was tested. Bcl-2 and p53 afforded similar protection against cell death on irradiation as that afforded by Bcl-XL and a poor outcome should be expected in patients treated with radiotherapy for TCC of the bladder expressing both Bcl-2 and p53 [21]. Hence those patients should not be offered the treatment on first place.

## Conclusion

Adjuvant radiotherapy for muscle invasive bilharzial related squamous cell carcinoma of the urinary bladder is a practice that should be considered for reevaluation due to its potential effectiveness and minor side effects. Moreover, Bcl-XL expression is a valuable tool for predicting the response to AR in those patients, hence detecting patients who are most likely to benefit from the adjuvant treatment.

## Competing interests

The authors declare that they have no competing interests.

## Authors' contributions

AA shared in conception and design of the study, acquisition of data and drafting the manuscript; DH shared in acquisition of data, interpretation of data and drafting the manuscript; EE shared in acquisition of data, interpretation of data and critical revision of the manuscript; EM responsible about laboratory evaluation of the patients and shared in drafting the manuscript; MEA responsible about radiotherapy and shared in drafting the manuscript; NK responsible about histopathologic evaluation of the patients and shared in drafting the manuscript; HH shared in conception and design of the study and drafting the manuscript; MAA shared in conception and design of the study and drafting the manuscript; KH shared in analysis and interpretation of data, involved in drafting the manuscript and critical revision of the manuscript. Each author has participated sufficiently in the work, takes public responsibility for appropriate portions of the content, have given final approval of the version to be published.

## Pre-publication history

The pre-publication history for this paper can be accessed here:

http://www.biomedcentral.com/1471-2407/11/16/prepub
